# Comparing the effects of saffron, lippia, and saffron-lippia combination on anxiety among candidates for coronary angiography

**DOI:** 10.22038/AJP.2021.18077

**Published:** 2021

**Authors:** Kobra Soheilipur, Mohammad Reza Khazdair, Seyyed Ali Moezi, Gholamhossein Mahmoudirad

**Affiliations:** 1 *Faculty of Nursing and Midwifery, Birjand University of Medical Sciences, Birjand, Iran *; 2 *Cardiovascular Diseases Research Center, Birjand University of Medical Sciences, Birjand, Iran*

**Keywords:** Coronary angiography, Anxiety, Saffron, Lippia citriodora, Crocus sativus

## Abstract

**Objective:**

Coronary artery disease (CAD) is among the most prevalent diseases in the world, and its severity is usually assessed through coronary angiography (CA). Anxiety is the most prevalent problem before angiography. The aim of this study was to evaluate *Crocus sativus *(saffron) and *Lippia citriodora *Kunth (lippia) extracts and saffron-lippia extract combination on anxiety among the candidates for CA.

**Materials and Methods:**

This double-blind randomized placebo-controlled trial in four month was conducted on 120 angiography candidates in Valiasr hospital, Birjand, Iran. The random allocation was doing through block randomization to saffron, lippia, saffron-lippia, and placebo groups; four hours before angiography, participants in these groups respectively received a single dose of saffron extract capsule (40 mg), lippia extract capsule (40 mg), saffron-lippia extract capsule (20 mg saffron and 20 mg lippia), and lactulose capsule (40 mg). Participants’ anxiety was assessed before, thirty minutes after, and three hours after the intervention via Spielberger State-Trait Anxiety Inventory.

**Results:**

The mean scores of state, trait, and total anxiety in the saffron group reduced significantly (p<0.05), while the mean scores in the other groups did not change significantly, except for the mean score of trait anxiety in the saffron-lippia group which decreased significantly (p=0.05). Therefore, after the intervention, the mean scores of state, trait, and total anxiety in the saffron group were significantly lower than the other groups (all, p<0.05).

**Conclusion:**

This study suggests that the oral use of a single-dose of 40 mg saffron extract is effective in alleviating anxiety among the candidates for CA.

## Introduction

Coronary artery disease is among the most prevalent diseases worldwide, so that the World Health Organization referred to it as the epidemic of the modern era. It is so common that most people know its symptoms, chiefly chest discomfort and dyspnea (Edition, 2006 ▶). The disease is a leading cause of mortality worldwide. The American Heart Association reported that one third of all deaths are related to coronary artery disease (Mohammad Hassani et al., 2010 ▶). Around 50% of all deaths in Iran are also due to this disease (Moeini et al., 2010 ▶) 

The severity of coronary artery disease is usually assessed through invasive diagnostic procedures such as coronary angiography (CA) or cardiac catheterization (Lewis et al., 2014 ▶). During CA, a plastic catheter is inserted into an artery or vein under local anesthesia and is guided towards the heart. The catheter is placed in the right or the left coronary arteries under fluoroscopy and then, a radiopaque agent is injected simultaneously with radiographic imaging (Edition, 2006 ▶). Around 16000–18000 coronary angiography are performed in Iran annually (Sadephy, 2007 ▶).

Invasive procedures usually cause patients different levels of anxiety. For instance, a study showed that 82% of CA candidates suffer from pre-CA anxiety (Heikkilä et al., 1998 ▶). Anxiety is the most common psychological problem worldwide and the most common psychological reaction to new changes and situations (Hanifi et al., 2012 ▶). There are two general types of anxiety, namely trait (or latent) and state (or manifest) anxiety. Trait anxiety refers to personality traits which cause some people to be more likely to experience anxiety. On the other hand, state anxiety happens in certain situations or in response to changes (Hanifi et al., 2012 ▶; Tahmasbi et al., 2012 ▶). The major factors behind CA-related anxiety are lack of knowledge, unfamiliar and strange CA environment, concern over potential CA complications, and intolerance to pre- and post-CA care services (Hanifi et al., 2012 ▶). 

Anxiety can cause different complications. For instance, CA-related anxiety increases myocardial oxygen demand, reduces blood flow to the myocardium, and thus, causes chest pain and cardiac arrhythmias. It also increases heart rate, blood pressure, and the risk of cardiac arrhythmias and reduces blood flow to wounds through stimulating sympathetic nervous system. Long-term anxiety postpones wound healing, increases the risk of infection, alters immune response, causes fluid and electrolyte imbalance, and disturbs sleep (Hanifi et al., 2012 ▶). Anxious patients suffer fatal cardiac events 4–6 times more than others. Therefore, effective anxiety management is of great importance, particularly among the candidates for invasive cardiac procedures (Shamsizadeh, 2013 ▶). Anxiety alleviation and management before invasive procedures reduce the length of procedures and the risks of fatal problems (such as arrhythmias) and help healthcare professionals establish correct diagnosis (Tahmasbi et al., 2012 ▶).

There are different pharmacological and non-pharmacological therapies for anxiety. The most common pharmacological therapy employs benzodiazepine agents. These agents have strong anxiolytic effects. Yet, even their short-term use is associated with different side effects such as somnolence, confusion, and respiratory problems. These side effects may be severer among older people (St Charles's Hospital).Non-pharmacological therapies for anxiety include patient education, behavioral therapy, and complementary and alternative medicine therapies (Tahmasbi et al., 2012 ▶). The World Health Organization reported the increasing prevalence of using complementary and alternative medicine therapies because of their high public acceptability, holistic view toward health and human, and easy applicability (World Health Organization, 1978 ▶). Iranian Traditional Medicine is among complementary and alternative therapies. It is mainly based on herbal medicine (Bakhtiari, 1389 ▶). There are different medicinal plants in Iranian Traditional Medicine for inducing and promoting tranquility. For example, saffron and *Lippia citriodora *have been used for many years as tranquilizing tea and aromatic spices.

Saffron, scientifically known as *Crocus sativus*, is a small perennial bulbous herbal plant from the Iridaceae family (Hoshyar et al., 2012 ▶; Khazdair et al., 2015 ▶). It is widely harvested in central and eastern parts of Iran. The active components of saffron are crocin, crocetin, and safranal (Liakopoulou-Kyriakides and Kyriakidis, 2002 ▶). Saffron has different pharmacological effects and is considered as an effective herbal medicine because only small amounts of its extract powder (30–100 mg/daily) can produce considerable pharmacological effects (Kianbakht, 2008 ▶). In Iranian Traditional Medicine, saffron extract and tincture are used to facilitate digestion, promote tranquility and expectoration, and manage liver and gallbladder disorders, muscular cramp and spasm, abdominal distension, insomnia, infections, cancers and respiratory disorders (Kianmehr and Khazdair, 2020 ▶; Mortazavi Moghaddam et al., 2020 ▶; Shahhoseini et al., 2011 ▶). Moreover, like diazepam, it has muscle relaxant and antispasmodic effects (Ai et al., 1997 ▶; Mokhtari-Zaer et al., 2015 ▶). Previous studies proved the anxiolytic effects of saffron in rats (Pitsikas et al., 2008 ▶), and its antispasmodic effects in mice (Hosseinzadeh and Noraei, 2009 ▶). A study on patients with anxiety also showed that 50 mg saffron capsule taken twice daily for twelve weeks, significantly alleviated anxiety (Mazidi et al., 2016 ▶).

Another medicinal plant with tranquilizing effects in Iranian Traditional Medicine is (*Lippia citriodora *Kunth) from the Verbenaceae family. Lippia (also known as *Aloysia citrodora*, lemon verbena, and beebrush) is a perennial shrub with a height of 1.5–2.5 meters and simple acuminate leaves. It is found in central and western areas of Iran (Shahhoseini et al., 2011 ▶), and is marketed as dried leaves and as distillates (Essence). Lippia tea has tranquilizing, anticonvulsant, sedative, and antihypertensive effects and reduces palpitation. Lippia is among the most commonly used products and treatments for insomnia and anxiety (Shahhoseini et al., 2011 ▶). An animal study revealed that like diazepam, lippia extract (1–10 mg per kg of body weight) had positive effects on the A receptors of gamma aminobutyric acid which can reduce anxiety (Fukui et al., 2011 ▶). A review study on different genera of lippia also concluded that lippia is a tranquilizer and antispasmodic agent (Ragone et al., 2010 ▶).

To the best of our knowledge, there is limited information about the effects of saffron and lippia on anxiety among CA candidates. This study was designed and undertaken to bridge this gap. The aim of the study was to evaluate the pure effects of saffron and lippia extracts and the effects of saffron-lippia extract combination on anxiety among CA candidates.

## Materials and Methods

This double-blind randomized placebo-controlled trial was conducted in 2017. Patients who referred to the angiography unit of Valiasr hospital, Birjand, Iran, were conveniently recruited and randomly allocated through block randomization to saffron, lippia, saffron-lippia, and placebo groups. Inclusion criteria were an age of 50–75 years old, a non-emergency angiography, hospitalization 24 hr before angiography, no /history of anxiety or anxiolytic agents use, no current use of chemical anxiolytic agents, and no known sensitivity to saffron and lippia. Participants who voluntarily withdrew from the study were excluded. This study was approved by the Ethics Committee of Birjand University of Medical Sciences, Birjand, Iran, and registered in the Iranian Registry of Clinical Trials (registration number: IRCT2015102524680N1). Clear explanations were provided to all participants about the aim of the study. Participants were ensured of voluntary participation in the study and confidential management of the study data. 

**Sample size calculation**

Using the results of a previous study (Shahmansouri et al., 2014 ▶), thirty patients were determined to be necessary for each study group, 120 in total, using the following formula:

$n=\frac{{\left(Z_{1-\frac{\alpha }{2}}+Z_{1-\beta }\right)}^{2}(S_{1+}^{2}S_{2}^{2})}{{\left(\mu_{2-}\mu_{1}\right)}^{2}}=\frac{{\left(1.96+0.68\right)}^{2}({\left(5.5\right)}^{2}+{\left(4.39\right)}^{2}}{{\left(11.65-8.10\right)}^{2}}=27.39\mathrm{\sim 30}$

**Instrument**

Study data were collected via Spielberger State-Trait Anxiety Inventory (STAI). This inventory contains forty items. Items 1–20 assess state anxiety and items 21–40 assess trait anxiety. Positively-worded items are scored 1–4, while negatively-worded items are scored 4–1. The possible scores of total, state, and trait anxiety are 40–160, 20–80, and 20–80. 

Reliability and validity of this inventory were confirmed in the study of Dehghan Nayeriby (Cronbach’s alpha 0.94) and the study of Bayaniby (Cronbach’s alpha 0.92) in Iran (Bayani 2008 ▶; Dehghan-nayeri and Adib-Hajbaghery, 2011 ▶).

**Preparation of the extracts**

To prepare 15 mg liquid extract of saffron and lippia, 100 mg saffron and lippia stigma powder were grounded. The resulting powder passed through percolation device, and extracts were taken in three stages using sterile water. Then, pure deionized water was added and the combination was kept in a sealed container for 48 hr. This mixture was stirred several times during the 48-hrtime period so as to let the extract be produced. As the extract was prepared, it was filtered and re-rinsed with ionized water. After adding the powder and solvent, when the liquid began to drip out, the bottom valve of percolator was closed and the percolator’s opening was covered for 48 hr; afterwards, the bottom valve was opened, letting the solvent drip out slowly in drops.

The extract collected from the percolation was poured into a vacuum distillation machine to be concentrated under 35-40°C heat.

**Intervention**

**Figure 1:**
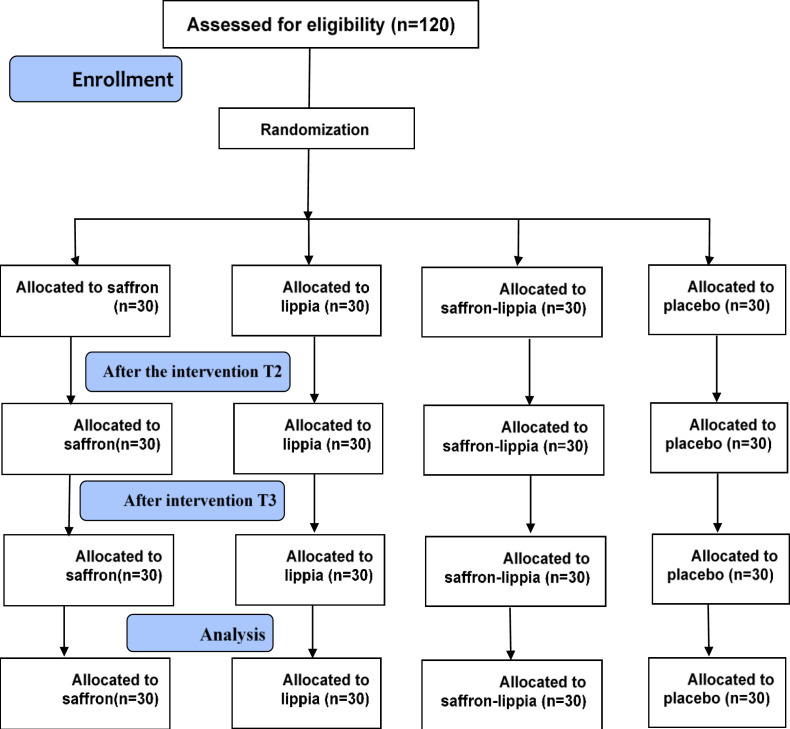
Flowchart of the study selection of patients

CA candidates in the saffron, lippia, saffron-lippia, and placebo groups were respectively treated with saffron capsules (contained 40 mg saffron extract powder), lippia capsules (contained 40 mg lippia extract powder), saffron-lippia capsules (contained 20 mg saffron extract powder and 20 mg lippia extract powder), and lactulose capsules (contained 40 mg lactulose powder), (Figure 1). Dosage of the drugs were prepared according to the previous studies for saffron (Moazen-Zadeh et al., 2018 ▶), lipia (Razavi et al., 2017 ▶), and placebo (Basiri-Moghadam et al., 2016 ▶). Capsules were produced by Kerman Faculty of Pharmacy, Kerman, Iran. All steps of extract production (namely extraction, distillation, and drying) were taken under sterile conditions and under the supervision of a pharmacist.

Four hours before angiography, participants were asked to complete STAI (the T1 measurement point). Then, each of them was asked to randomly select one card from the four cards labeled A (saffron group), B (lippia group), C (saffron-lippia group), or D (placebo group). Based on the label on the selected card, the participant was allocated to one of the groups and then, was asked to take one dose of the allocated capsule. Finally, all participants recompleted STAI thirty minutes and 3hrafter taking the capsule (the T2 and T3 measurement points, respectively).

Collected data were entered into the IBM SPSS software (v. 20) and described via the measures of descriptive statistics such as mean and standard deviation. Moreover, the one-way analysis of variance (ANOVA) and the repeated measures ANOVA as well as the Kolmogorov-Smirnov, Chi-square, Fisher exact, Bonferroni’s, and Tukey’s range tests were conducted for data analysis. 

## Results

**Figure 2:**
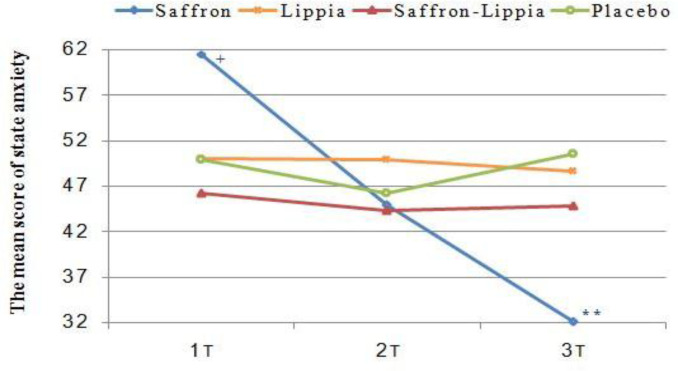
The variations of the mean score of state anxiety in all groups over time. Data were presented as mean±SD. Despite the random allocation, there was a significant difference between the anxiety score of the saffron group and other groups in the pre-test that controlled by mean differences of the scores each group. +p<0.05, comparison of the saffron with the other groups and **p<0.001, comparison of the saffron with the placebo group

**Figure 3:**
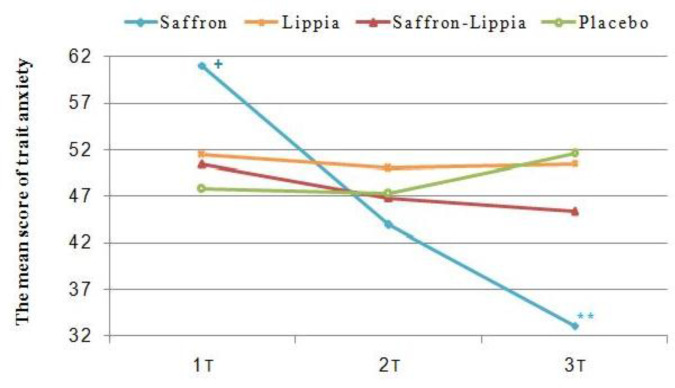
The variations of the mean score of trait anxiety in all groups over time. Data were presented as mean±SD. +p<0.05, comparison of the saffron with the other groups and **p<0.001, comparison of the saffron with the placebo group

**Figure 4:**
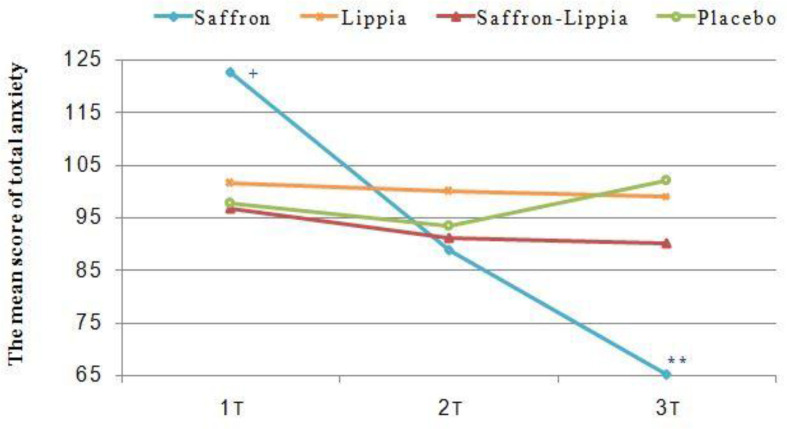
The variations of the mean score of total anxiety in all groups over time. Data were presented as mean±SD. +p<0.05, comparison of the saffron with the other groups and **p<0.001, comparison of the saffron with the placebo group

The mean age of the 120 participating CA candidates was 61.33 years old. Most participants were male (53.3%) and married (88.3%) (Figures 2-4). The four study groups did not significantly differ from each other respecting participants’ age, gender, and marital status (Table 1).

**Table 1:** Comparisons of the groups concerning participants’ age, gender, and marital status

**Groups**	**Gender, N (%)**		**Age ( year )**	**Marital status, N** **(%)**	
	Female	Male	Mean±SD	Single	Married
Saffron	12 (40)	18 (%60)	60.97±7/71	1 (3.3)	29 (96.7)
Lippia	16 (53.3)	14 (46.7)	62.03±8/58	4 (13.3)	26 (86.7)
Saffron-lippia	14 (46.7)	16 (53.3)	60.97±5/46	4 (13.3)	26 (86.7)
Placebo	14 (46.7)	16 (53.3)	61.33±5/30	5 (11.7)	25 (88.3)
p value	*0/80		**0/92	***0/92	

Data are expressed as n (%) or mean±SD. *: The results of x2. **: The results of the one-way ANOVA. ***: The results of fisher exact test

The results of the one-way ANOVA illustrated significant differences among the groups respecting the pretest mean scores of state, trait, and total anxiety (p<0.001). The Tukey’srange post hoc test showed that all mean scores in the saffron group were significantly greater than the other groups (p<0.05). However, there were no significant differences among the groups regarding the mean scores of state, trait, and total anxiety thirty minutes after the intervention. Three hours after the intervention, the groups significantly differed from each other respecting the mean scores of state, trait, and total anxiety (p<0.001). The results of the Tukey’s range post hoc test illustrated that the mean scores of state, trait, and total anxiety in the saffron group were significantly less than the other groups (p<0.05; Table 2 and Figures 2-4).

The results of repeated measures ANOVA for the within-subject factor of time showed significant differences among the three measurement time points in the saffron group respecting the mean scores of state, trait, and total anxiety (p<0.001). The Bonferroni’s post hoc test revealed that the mean scores of state, trait, and total anxiety at T2 were significantly less than T1 and the mean scores at T3 were significantly less than T1 and T2 (p<0.001; Table 2 and Figures 2–4).

The repeated measures ANOVA for the within-subject factor of time also showed that in the lippia and the placebo groups, there were no significant differences among the three measurement time points respecting the mean scores of state, trait, and total anxiety (p>0.05; Table 2 and Figures 2–4).

The results of the repeated measures ANOVA for the within-subject factor of time also indicated that in the saffron-lippia group, the differences among the three measurement time points respecting the mean scores of total and state anxiety were not statistically significant. However, in this group, there was at least one significant difference among the three measurement time points respecting the mean score of trait anxiety (p=0.05). The Bonferroni’s post hoc test indicated that the difference was between T1 and T3, so that the mean score of trait anxiety at T3 was significantly less than T1 (p=0.02; Table 2 and Figures 2-4). Respecting the differences between each two measurement time points, the results of the one-way analysis of variance indicated significant differences among the groups in terms of the T1-T2, T1–T3, and T2–T3 mean differences of state, trait, and total anxiety (p<0.001). The results of the Tukey’s range post hoc test showed that T1-T2, T1–T3, and T2–T3 mean differences of state, trait, and total anxiety in the saffron group were significantly greater than the other groups. Also, the mean changes of latent anxiety score T1-T2 in patients mixed with lippia and saffron were significantly higher than the control group (p=0.03), and mean changes in overt anxiety score T2–T3 in the placebo group were significantly higher than lippia extract group (p<0.05). The mean changes in latent anxiety and total anxiety score T2–T3 in placebo group were significantly higher than saffron-lippia patients and lippia-treated group (p<0.05; Table 3). Will be reminded that increasing in mean changes anxiety score in placebo group were in direction to increasing of anxiety score.

**Table 2:** Within-group and between-group comparisons concerning the mean scores of state, trait, and total anxiety

Anxiety	Time Group	T1	T2	T3	p value^	p value^†^		
						T1-T2	T1-T3	T2-T3
State	Saffron	61.53±13.86	44.93±7.97	32.07±9.26	<0.001	<0.001	<0.001	<0.001
	Lippia	50.07±8.09	49.90±6.67	48.63±5.89	0.47	0/47	0/47	0/47
	Saffron-lippia	46.20±14.99	44.33±1395	44.77±12.34	0.61	0/61	0/61	0/61
	Placebo	49.90±13.11	46.20±15.69	50.50±14.04	0.14	0/14	0/61	0/61
	p value*	<0.001	0.26	<0.001	—	—	—	—
Trait	Saffron	61.10±11.42	43.97±7.05	33.13±10.53	<0.001	<0.001	<0.001	<0.001
	Lippia	51.53±8.51	50.07±7.28	50.50±6.39	0.60	0/60	0/60	0/60
	Saffron-lippia	50.43±10.95	46.87±12.54	45.43±11.70	0.05	0/09	0.02	0/25
	Placebo	47.80±13.84	47.30±14.76	51.63±12.49	0.10	0/10	0/10	0/10
	p value*	<0.001	0.20	<0.001	—	—	—	—
Total	Saffron	122.63±24.77	88.90±14.40	65.20±19.17	<0.001	<0.001	<0.001	<0.001
	Lippia	101.60±15.54	99.97±12.87	99.13±11.42	0.59	0/59	0/59	0/59
	Saffron-lippia	96.63±24.56	91.20±24.61	90.20±22.75	0.12	0/12	0/12	0/12
	Placebo	97.70±25.89	93.50±30.02	102.13±26.13	0.13	0/13	0/12	0/12
	p value*	<0.001	0.23	<0.001	—	—	—	—

Data were presented as mean±SD. *: The one-way ANOVA; ^: The repeated measures ANOVA; †: The Bonferroni’s post hoc test; T1: Before the intervention; T2: Thirty minutes after the intervention; T3: Three hours after the intervention

**Table 3:** Comparisons of the mean differences the scores of anxiety in the four groups

Anxiety	Group Time	Saffron**	Lippia	Saffron-lippia**	Placebo	p value ANOVA*
State	T1-T2	-16.60	-0/17	-1/78	-3/70	<0/001
	T1-T3	-29/47	-1/43	-1/43	0/60	<0/001
	T2-T3	012/87	-1/27	0/43	4/30	<0/001
Trait	T1-T2	-17/13	-1/47	-3/57	-0/50	<0/001
	T1-T3	-27/97	-1/03	-5	3/83	<0/001
	T2-T3	-10/73	0/43	-1/43	4/33	<0/001
Total	T1-T2	-33/73	-1/63	-5/43	-4/20	<0/001
	T1-T3	-57/43	-2/47	-6/43	4/43	<0/001
	T2-T3	-23/70	-0/83	-1	8/63	<0/001

T1: Before intervention; T2: Thirty minutes after intervention; T3: Three hours after intervention, T1-T2: scores of T1 minus T2, T1-T3: scores of T1 minus T3, T2-T3: scores of T2 minus T3, *: The one-way ANOVA; T1: Before intervention; T2: Thirty minutes after intervention; T3: Three hours after intervention, **: The results of the Tukey’s range post hoc test showed that T1-T2, T1–T3, and T2–T3 mean differences of state, trait, and total anxiety in the saffron group were significantly greater than the other groups (p<0.001).

## Discussion

This study evaluated the pure effects of saffron and lippia extracts and the effects of saffron-lippia extract combination on anxiety among CA candidates. In the current study, participants did not report any side effect for saffron or lippia. The results of a clinical trial on crocin tablet showed that this component of saffron is safe herbal product in therapeutic doses (Bostan et al., 2017 ▶). Similar studies on saffron tablets also did not report important clinicall toxicity in healthy volunteers (Bostan et al., 2017 ▶). It was shown that treatment with the aqueous extract of *Lippia citriodora* leaves was well tolerated in daily IP injection at doses up to 200 mg/kg in mice and rats for a period of 21 days and did not produce any toxicity (Etemad et al., 2016 ▶).

Our study findings revealed the effectiveness of saffron extract in alleviating anxiety. Similarly, an animal study in mice showed that 0.56 mg/kg of saffron extract had the same anxiolytic effects as 3 mg/kg of diazepam and 30 mg/kg of thiopental sodium (Hosseinzadeh and Noraei, 2009 ▶). Another study reported that twenty-minute saffron aromatherapy significantly alleviated anxiety among women with dysmenorrhea (Fukui et al., 2011 ▶). Moreover, a study found that oral use of 50 mg saffron capsules twice/daily for twelve successive weeks significantly reduced anxiety symptoms among patients with moderate to severe anxiety disorders (Mazidi et al., 2016 ▶). A review study also noted that saffron can be effective in alleviating anxiety (Pandey et al., 2013). Another review study considered saffron as an option for the management of anxiety and schizophrenia (Hosseinzadeh and Noraei, 2009 ▶). In Iranian Traditional Medicine, saffron is considered to have tonic, relaxant, and antispasmodic effects (Javadi et al., 2013 ▶).

The medicinal effects of saffron are attributed to its crocin and safranal components. An animal study showed that crocin has relaxant and anxiolytic effects, while safranal only has anxiolytic effects. That study concluded that crocin can exert antidepressant effects through affecting the dopaminergic system and inhibiting norepinephrine reuptake. Moreover, safranal was reported to affect serotonin , cause changes in the lipid membrane structures of gamma aminobutyric acid receptors and cause changes in chloride channels (Hosseinzadeh and Noraei, 2009 ▶). These effects are almost similar to the effects of benzodiazepines which reduce anxiety through affecting the limbic system and active metabolites as well as the metabolism and the receptors of gamma aminobutyric acid in the brain. 

Our findings also indicated no significant changes in anxiety level in the lippia group across the three measurement time points. However, a review study reported traditional use of lippia for promoting tranquility in most Central American and North African countries (Pascual et al., 2001 ▶). An earlier animal study in mice and rats also showed that subcutaneous injection of 1–10 mg/kg of lippia extract had the same tranquilizing effects as diazepam and flumazenil (Ragone et al., 2010 ▶). Another animal study in rats also found that intra-peritoneal injection of 200 mg/kg of lippia extract significantly alleviated anxiety (Eidi et al., 2014 ▶). The contradiction between our findings and the findings of these two animal studies can be attributed to the differences between animal and human samples in the studies. Further human studies are needed to provide more credible evidence regarding the anxiolytic effects of lippia.

We also found the insignificant effects of saffron-lippia combination on pre-CA anxiety. This finding may be due to the low dose of saffron (20 mg) in the combination. An earlier study compared 40 and 80 mg doses of saffron and reported that the dose 80 mg of saffron was more effective than the dose 40 mg in alleviating depression (Moosavi et al., 2014 ▶).

This study suggests that the oral use of a single-dose of 40 mg saffron extract is effective in alleviating anxiety among the candidates for CA, while lippia extract and saffron-lippia extract combination had no significant effects on their anxiety. Of course, further studies are still needed to produce adequate evidence concerning the effects of saffron and lippia extracts on different types of anxiety among different populations of patients.
